# One step synthesis of efficient red emissive carbon dots and their bovine serum albumin composites with enhanced multi-photon fluorescence for in vivo bioimaging

**DOI:** 10.1038/s41377-022-00798-5

**Published:** 2022-04-27

**Authors:** Huiqi Zhang, Gang Wang, Zhiming Zhang, Josh Haipeng Lei, Tzu-Ming Liu, Guichuan Xing, Chu-Xia Deng, Zikang Tang, Songnan Qu

**Affiliations:** 1 Joint Key Laboratory of the Ministry of Education, Institute of Applied Physics and Materials Engineering, University of Macau, Macau, 999078 China; 2 Institute of Translational Medicine, Faculty of Health Sciences, University of Macau, Macau, 999078 China; 3 MoE Frontiers Science Center for Precision Oncology, University of Macau, Macau, 999078 China; 4 Cancer Center, Faculty of Health Sciences, University of Macau, Macau, 999078 China

**Keywords:** Imaging and sensing, Nanoparticles

## Abstract

Efficient red emissive carbon dots (CDs) in aqueous solutions are very scarce for high performance bioimaging applications. In this work, we report a one-step solvothermal treatment to synthesize pure red emissive CDs (FA-CDs) from citric acid and urea in formic acid without complicated purification procedures. Photoluminescence quantum yield (PLQY) of 43.4% was observed in their dimethyl sulfoxide solutions. High PLQY up to 21.9% in aqueous solutions was achieved in their bovine serum albumin (BSA) composites (FA-CDs@BSA) with significantly enhanced multi-photon fluorescence. The strong surface electron-withdrawing structure of FA-CDs caused by the high content of C = O groups contributes for their pure red emission. Owing to the significantly enhanced single and multi-photon red fluorescence and enlarged particle sizes after composing with BSA, in vivo tumor imaging and two-photon fluorescence imaging of blood vessels in mouse ear have been realized via intravenous injection of FA-CDs@BSA aqueous solutions.

## Introduction

Carbon dots (CDs) are a kind of zero-dimensional carbonaceous nanomaterial, which are composed of sp^2^- and/or sp^3^-hybridized carbon domains, and decorated by surface functionalities. Due to their ultra-small size (usually less than 10 nm), simple synthesis, low toxicity, well-documented biocompatibility, and outstanding luminescent properties, CDs have attracted wild applications, particularly in bioimaging and biomedical fields^1,2^. Noninvasive in vivo fluorescence (FL) imaging calls for an ideal working spectral window in red to NIR range of 650–1450 nm^3^. Therefore, CDs with strong emissions in red to NIR region in aqueous solutions are highly desired.

Until now, efficient blue and green emissions with photoluminescence quantum yield (PLQY) higher than 60% have been reported in several CDs systems^4,5^, while efficient pure red emissive CDs are still scarce. For example, Jiang and coworkers reported red emissive nitrogen- and fluorine-doped CDs from citric acid, urea, and ammonium fluoride by solvothermal treatment in dimethylformamide (DMF), followed by post alkali treatment^6^. These CDs exhibited red emission with PLQY of 9.8% in aqueous solutions. Zhang and coworkers prepared p-phenylenediamine based CDs by solvothermal method^7^. The red emissive CDs can be separated through silica gel column chromatography with PLQY of 31.4% in ethanol. Fan and coworkers reported red emissive CDs with large amino acid from 1,4,5,8-tetraminoanthraquinone and citric acid through solvothermal method followed by purification through silica gel column chromatography^8^. PLQY of 10.1% was measured in their DMF solutions. It can be seen that the reported efficient red emissive CDs were fabricated in multiple steps including complicated purification procedures, such as silica-gel column chromatography. More seriously, high PLQYs of the red emissions from these CDs were always obtained in their organic solutions^9,10^, but be significantly decreased in water^11–13^, which greatly hinder their application for in vivo bioimaging in aqueous system.

It has been demonstrated that surface chemical structures play an important role in the luminescence properties of CDs^14,15^. In our previous work, we reported orange emissive CDs with large-sized conjugated sp^2^-domain through increasing the carbonization processes between citric acid and urea under solvothermal treatment in DMF^16^. Enhanced PLQY of 46% in ethanol was realized through surface metal cation-functionalizing of these CDs, but they exhibited a low PLQY of 6% in water. We further demonstrated solvents with electron-rich (S = O/C = O) groups, such as dimethyl sulfoxide (DMSO), DMF, N-methyl-2-pyrrolidone(NMP), can facilitate the enhancement of the red emissions from CDs^17^. It can be inferred that increasing the surface electron-rich groups is beneficial for pure red emissive CDs with high PLQY.

In this work, we developed a one-step solvothermal treatment to synthesize pure red emissive CDs from citric acid and urea in formic acid (FA) without complicated purification procedures. PLQY of 43.4% was observed in their DMSO solutions. Comparing the CDs synthesized from citric acid and urea in different organic solvents under the same solvothermal treatments, the FA solvothermal prepared CDs (FA-CDs) contained the highest content of C = O groups on their surface, resulting in uniform strong electron-withdrawing surface structure, which account for their pure red emission. The red emission from FA-CDs was relatively weak in water but can be effectively enhanced after composing with bovine serum albumin (BSA) with PLQY up to 21.9% in their composite (FA-CDs@BSA) aqueous solutions. The prepared FA-CDs and FA-CDs@BSA exhibit low cytotoxicity and renal excretion. Moreover, the FA-CDs@BSA can accumulate in the tumor tissue through the blood circulation with a clearly red FL tumor imaging in a long observation time window (>6 h). Furthermore, significantly enhanced two- and three-photon red emissions were observed in FA-CDs@BSA aqueous solutions. Two-photon FL imaging of mouse ear vessels has also been achieved via intravenous injection of FA-CDs@BSA aqueous solutions. To our knowledge, this is the first time of realizing in vivo two-photon FL imaging in mammals based on CDs^13,18–21^.

## Results

FA-CDs were synthesized from citric acid and urea by a one-step solvothermal treatment in FA. Specifically, citric acid (2 g), urea (4 g), and 20 mL FA were reacted at 160 °C for 4 h under solvothermal condition. The reacted dark red-brown solution was added to 40 mL ethanol, shaken well, and then centrifuged at 10,000 r min^−1^ for 5 min and repeated 2–3 times. The precipitate was freeze-dried to obtain the black product of FA-CDs.

**Fig. 1:**
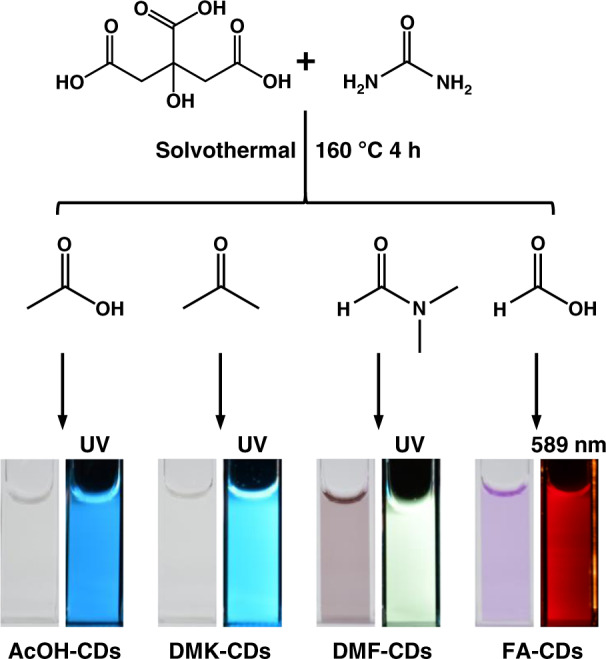
The preparation routes of AcOH-CDs, DMK-CDs, DMF-CDs and FA-CDs by solvothermal treatments. Bottom images show the photographs of AcOH-CDs, DMK-CDs, DMF-CDs and FA-CDs aqueous solutions in bright field (left) and fluorescence (FL) field (right)

To investigate the effect of solvents in the solvothermal treatments on the morphologies, chemical structures and optical properties of CDs, acetic acid (AcOH), acetone (DMK), and DMF were chosen as solvents to prepare CDs under the same solvothermal treatments, which were AcOH-CDs, DMK-CDs, and DMF-CDs, respectively, as shown in Fig. 1.

**Fig. 2:**
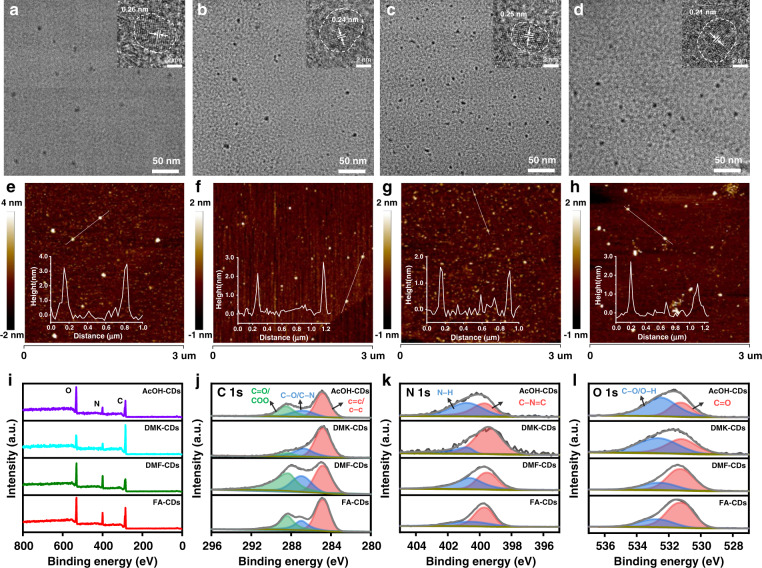
The morphologies and chemical structures of AcOH-CDs, DMK-CDs, DMF-CDs and FA-CDs **a**–**d** TEM and HRTEM (insets) images of AcOH-CDs, DMK-CDs, DMF-CDs and FA-CDs. **e**–**h** AFM images of AcOH-CDs, DMK-CDs, DMF-CDs and FA-CDs, the insets at bottom left show the height profiles along the lines, respectively. **i** The full survey of XPS spectra of AcOH-CDs, DMK-CDs, DMF-CDs and FA-CDs. High-resolution XPS spectra of **j** C 1 s, **k** N 1 s, **l** O 1 s were deconvolved into several peaks labeled with different colors

The morphologies of the four samples were characterized using transmission electron microscopy (TEM) and atomic force microscopy (AFM). As seen in the TEM images (Fig. 2a–d), these samples show similar average particle diameters of about 5 nm with uniform dispersion. Similar well-resolved lattice fringes with an interlayer spacing of 0.26, 0.24, 0.25, and 0.21 nm can be observed in their high-resolution TEM (HRTEM) images, indicating all these samples exhibit similar graphite-like core structures^22–25^. The particle sizes of AcOH-CDs, DMK-CDs, DMF-CDs, FA-CDs from TEM and AFM images (Fig. 2e–h) are similar in the range of 1–7 nm.

X-ray photoelectron spectroscopy (XPS) was carried out to investigate the chemical structures of the four samples. The full survey of the XPS spectra of the four samples reveal the presence of C, N, O, as shown in Fig. 2i. The atomic content in the four samples is shown in Table 1. It can be seen that DMK-CDs, AcOH-CDs and DMF-CDs contain the highest carbon, oxygen and nitrogen content, respectively. The high resolution C 1 s, N 1 s, and O 1 s XPS spectra of the four samples reveal the presence of C = C/C − C (284.8 eV), C − N/C − O (286.8 eV), and C = O/COO (288.4 eV) bonds (from C 1 s XPS spectra); C − N = C (399.5 eV), N − H (400.7 eV)^26^ bonds (from N 1 s XPS spectra); and C = O (531.2 eV), C − O/O − H (532.6 eV) bonds (from O 1 s XPS spectra). By comparison, the C = O content in FA-CDs (17.43%) and DMF-CDs (16.26%) are much higher than that in AcOH-CDs (9.44%) and DMK-CDs (7.17%), while the N − H and C − O/O − H content in DMF-CDs (10.48% and 7.70%, respectively) are higher than that in FA-CDs (5.30% and 7.21%, respectively). Given the electron-withdrawing character of C = O groups and proton-donating character of −NH and −OH groups, the surface chemical structure of FA-CDs is more electron-withdrawing than that of DMF-CDs. According to morphology and XPS analysis, the FA-CDs exhibit the strongest electron-withdrawing surface structure among the four samples.

**Table 1:** The atomic content of C, N, O and the specific content of N-related and O-related chemical bonds of AcOH-CDs, DMK-CDs, DMF-CDs and FA-CDs in XPS spectra

	C	N	O	N		O	
				C − N = C	N − H	C = O	C − O/O − H
AcOH-CDs	59.26%	12.06%	28.68%	4.42%	7.64%	9.44%	19.24%
DMK-CDs	72.22%	9.58%	18.20%	7.84%	1.74%	7.17%	11.03%
DMF-CDs	55.61%	20.43%	23.96%	9.95%	10.48%	16.26%	7.70%
FA-CDs	59.84%	15.52%	24.64%	10.22%	5.30%	17.43%	7.21%

**Fig. 3:**
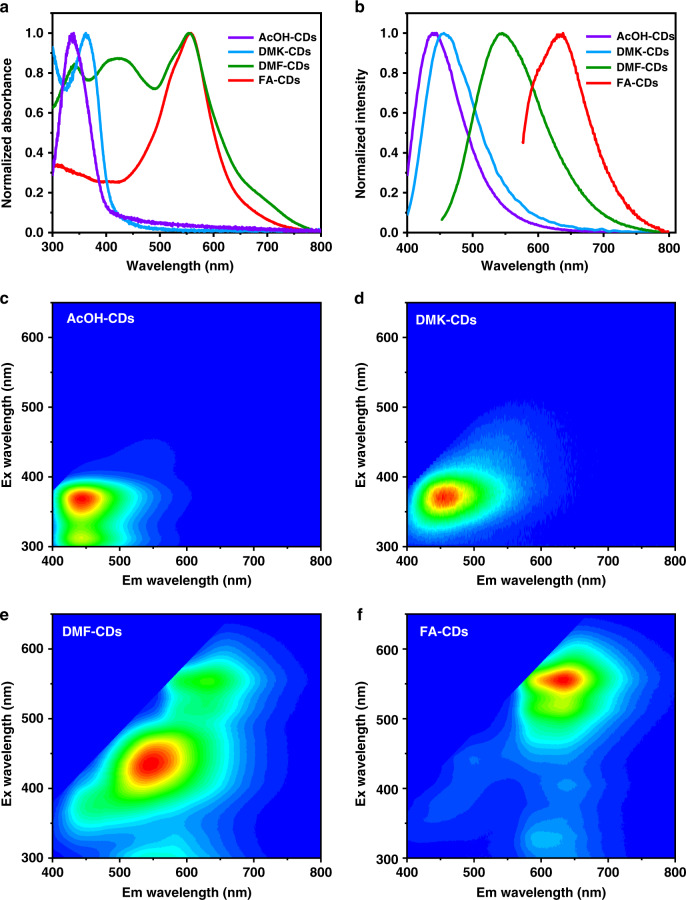
Absorption and emission spectra of AcOH-CDs, DMK-CDs, DMF-CDs and FA-CDs **a** Normalized UV–vis absorption spectra of AcOH-CDs, DMK-CDs, DMF-CDs and FA-CDs in dilute aqueous solutions. **b** The corresponding normalized maximum emission spectra of AcOH-CDs, DMK-CDs, DMF-CDs and FA-CDs in dilute aqueous solutions at 368, 375, 432 and 556 nm excitation, respectively. Excitation-emission maps of **c** AcOH-CDs, **d** DMK-CDs, **e** DMF-CDs and **f** FA-CDs in dilute aqueous solutions

The UV-visible absorption spectra of the four samples in aqueous solutions were measured (Fig. 3a). AcOH-CDs and DMK-CDs display absorption bands in UV region peaked at 337 nm and 361 nm, respectively. DMF-CDs exhibit a much broader absorption bands, with peaks at 340, 420, and 554 nm, while FA-CDs have a main absorption band at 556 nm. As shown in Fig. 3c, d, AcOH-CDs and DMK-CDs in aqueous solutions display similar single cyan luminescence centers in excitation-emission maps. DMF-CDs exhibit multiple luminescence centers covering from blue to red regions, with the main emission center in green region at 550 nm (Fig. 3e). In contrast, FA-CDs exhibit a main red emission center at 640 nm (Fig. 3f). Figure 3b shows the corresponding normalized maximum emission spectra of the four samples in aqueous solutions at 368, 375, 432, and 556 nm excitation, respectively. Considering the strongest electron-withdrawing surface structures of FA-CDs, it can be inferred the high content of C = O groups and low content of proton-donating −NH and −OH groups account for their pure red bandgap emission^27^.

**Fig. 4:**
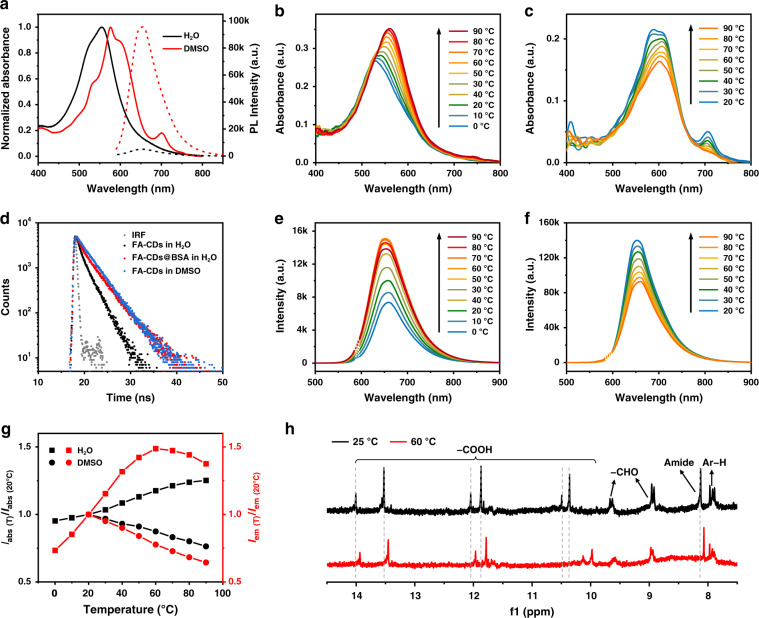
Absorption and emission spectra of FA-CDs in water and DMSO solutions **a** Absorption spectra (solid line) and FL spectra (dashed line) of FA-CDs in water and DMSO under 589 nm excitation. Temperature-dependent absorption spectra of FA-CDs in **b** water and **c** DMSO, respectively. **d** Luminescence decays of FA-CDs in water, FA-CDs@BSA (mass ratio of FA-CDs and BSA: 1:100) in water, and FA-CDs in DMSO monitored at 640 nm under 510 nm excitation (IRF = instrument response function). Temperature-dependent FL spectra of FA-CDs in **e** water and **f** DMSO, measured at 589 nm excitation. The concentration of FA-CDs is 0.01 mg mL^−1^. **g** Ratios of the maximum intensities of the absorption (I_abs_, black line) and FL (I_em_, red line) of FA-CDs in water (square) and DMSO (circular) at different temperatures to the maximum intensities of that at 20 °C. **h** ^1^H NMR spectra of FA-CDs in DMSO-d6 at 25 °C and 60 °C

To further investigate the influence of C = O, −OH and −NH groups on the surface environments, absorption and emission spectra of FA-CDs were measured in water and DMSO solutions, as shown in Fig. 4a. From protic water solvent to aprotic DMSO solvent, the main absorption band of FA-CDs was red-shifted from 556 nm to 576 nm with a shoulder band centered at 600 nm and an emerging small absorption band at 700 nm. The red emission from FA-CDs in water was relatively weak with PLQY of 6.0%, while its intensity can be 15-fold enhancement in DMSO with PLQY up to 43.4% (Table 2). DMSO is aprotic solvent with good deprotonation ability due to the S = O groups with strong electron-withdrawing ability. It had been demonstrated that deprotonation of the surface can enhance the surface electron-withdrawing environment of CDs, leading to redshifted absorption band, and enhanced emission intensity by inhibiting the energy dissipation of hydroxyl groups^17,28^. It can be inferred that the weak red emission from FA-CDs in water is due to the surface protonation caused by water molecules, while the redshifted absorption bands with enhanced red emission are due to the surface deprotonation caused by DMSO molecules. Figure 4d shows the FL decay curves of FA-CDs in water and DMSO solutions at 640 nm measured under excitation at 510 nm. FA-CDs exhibit a considerably longer average PL lifetime in DMSO than that in water, indicating much lower energy dissipation, which agrees well with their FL properties.

**Table 2:** PLQYs of FA-CDs and DMF-CDs in dilute aqueous solutions, FA-CDs@BSA and DMF-CDs@BSA at a mass ratio of FA-CDs/DMF-CDs and BSA with 1:200 in dilute aqueous solutions

	FA-CDs	DMF-CDs	FA-CDs@BSA	DMF-CDs@BSA
H_2_O	6.0%	1.5%	21.9%	7.3%
DMSO	43.4%	28.6%	–	–

Temperature-dependent absorption and emission spectra of FA-CDs in water and DMSO were further measured to investigate the affection of surface protonation and deprotonation processes on their optical properties (Fig. 4b, c, e, f). For FA-CDs in water (Fig. 4b, e), the absorption band gradually increased and red-shifted from 529 nm to 561 nm from 0 to 90 °C (Fig. 4b), while their red FL intensity first gradually increased until 60 °C and then slightly decreased upon further heating to 90 °C (Fig. 4e). In contrast, the absorption bands and red emission of FA-CDs in DMSO were gradually decreased upon heating to 90 °C (Fig. 4c, f). The ratios of the maximum intensities of absorption (I_abs_) and emission (I_em_) bands at different temperatures to the maximum intensities of that at 20 °C were given in Fig. 4g. The opposite temperature dependent optical behaviors of FA-CDs in water and DMSO can be explained by the protonation and deprotonation processes caused by water and DMSO molecules, respectively, through intermolecular hydrogen bonds between solvent molecules and surface groups on FA-CDs. In water, the hydrogen bonds between H_2_O molecules and the surface carbonyl groups on FA-CDs lead to surface protonation, which account for the quenched red FL. Upon increasing temperature, the hydrogen bonds were weakened, leading to the enhanced red-shifted absorption band and enhanced red emission at temperature below 60 °C. At the temperature higher than 60 °C, the gradually decreased FL intensity can be due to thermal vibration induced energy losses. In DMSO, the deprotonation process caused by hydrogen bonds between DMSO molecules and the surface proton-donating groups (−NH, − OH, −COOH) on FA-CDs can be weakened upon increasing temperature, leading to decreased absorption bands and red emissions. The hydrogen bonds between DMSO molecules and the surface proton-rich groups (−NH, − OH, −COOH) on FA-CDs can be further demonstrated by their temperature dependent ^1^H NMR spectra (Fig. 4h). The proton signals of Ar−H (7.8 ppm), amide (8.12 ppm), −CHO (8.5–10 ppm) and −COOH (10.36, 10.49, 11.87, 12.05, 13.53 and 14 ppm) were detected in the ^1^H NMR spectrum of FA-CDs in DMSO-d6 at 25 °C. Upon heating to 60 °C, the proton signals of the hydrogen bonds donor groups of amide and −COOH moved to 8.06 ppm and 9.97, 10.13, 11.78, 11.96, 13.45, 13.94 ppm, respectively, while the proton signals of Ar−H, −CHO were almost unchanged, indicating temperature dependent hydrogen bonds between DMSO molecules and the surface −NH and −COOH groups on FA-CDs.

Based on the results above, it can be concluded that the red bandgap emissions from CDs are not only determined by the particle sizes or the carbonization content of their cores, but also the surface electron-withdrawing environment. High content of surface electron withdrawing groups (C = O) on FA-CDs and DMF-CDs cause narrowed bandgaps, leading to redshifted absorption and emission bands. DMF-CDs contain more proton-donating −NH groups than FA-CDs, which weaken their surface electron-withdrawing environment, leading to non-sufficient redshifted emission centers with maximum emission in green light region. Although AcOH-CDs contain the highest oxygen content, while most of oxygen atoms are in the form of hydroxyl or ether groups and the content of C = O groups is much smaller, leading to much more weakened surface electron-withdrawing structure. By comparing the differences of the surface chemical structures of the four samples and the four solvent molecules in the solvothermal treatments, it can be inferred that the aldehyde group in FA and their acid nature play a significant role in the formation of high content of C = O groups and low content of −NH and −OH groups on the surface of FA-CDs. Although DMF molecules also contain aldehyde group, the weak alkaline nature of DMF solvent result in the prepared DMF-CDs containing much higher −NH groups than FA-CDs, which might be the reason for their different optical properties.

**Fig. 5:**
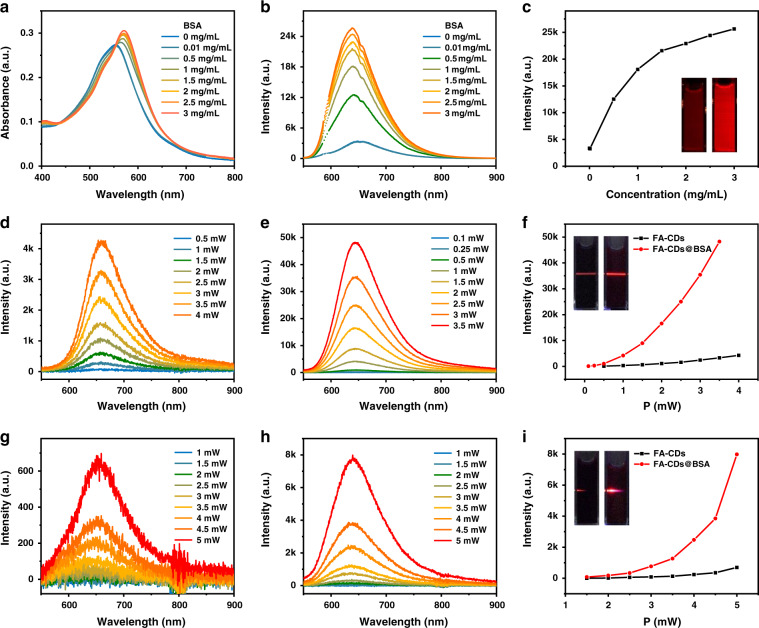
Single- and multi-photon FL spectra of FA-CDs and FA-CDs@BSA aqueous solutions **a** Absorption and **b** emission spectra of FA-CDs@BSA aqueous solutions at different composing ratios (FA-CDs: 0.01 mg mL^−1^, BSA: 0 to 3 mg mL^−1^). **c** Maximum FL intensities of FA-CDs@BSA aqueous solutions with varied BSA concentrations at the contained FA-CDs concentration of 0.01 mg mL^−1^. The insets show the FL images of FA-CDs (0.01 mg mL^−1^) (left) and FA-CDs@BSA (FA-CDs: 0.01 mg mL^−1^, BSA: 1 mg mL^−1^) (right) aqueous solutions under 589 nm excitation. Two-photon FL spectra of **d** FA-CDs (0.5 mg mL^−1^) and **e** FA-CDs@BSA (FA-CDs: 0.5 mg mL^−1^, BSA: 50 mg mL^−1^) in water at different powers of 1150 nm fs laser. **f** Maximum two-photon FL intensities under different power excitation. The insets show the photos of 0.5 mg mL^−1^ FA-CDs (left) and FA-CDs@BSA (FA-CDs: 0.5 mg mL^−1^, BSA: 50 mg mL^−1^) (right) in water under 1150 nm fs laser at 3.5 mW. Three-photon FL spectra of **g** FA-CDs and **h** FA-CDs@BSA in water at different powers of 1550 nm fs laser. **i** Maximum three-photon FL intensities under different power excitation. The insets show the photos of 0.5 mg mL^−1^ FA-CDs (left) and FA-CDs@BSA (FA-CDs: 0.5 mg mL^−1^, BSA: 50 mg mL^−1^) (right) in water under 1550 nm fs laser at 5 mW

To achieve enhanced red emission in aqueous solutions, BSA, a wildly used biocompatible protein, was selected to modify FA-CDs. 3 mL FA-CDs aqueous solutions (0.02 mg mL^−1^) was added to 3 mL BSA aqueous solutions with BSA concentrations of 0.02–6 mg mL^−1^. The mixed solutions were heated at 50 °C for 10 min to give rise FA-CDs and BSA composites (FA-CDs@BSA) with different composing ratios. Figure 5a, b shows the absorption and emission spectra of FA-CDs@BSA aqueous solutions at different composing ratios (FA-CDs: 0.01 mg mL^−1^). As increasing the BSA concentrations from 0 to 3 mg mL^−1^, the absorption bands of FA-CDs gradually increased and red-shifted from 556 nm to 571 nm, while their red emission exhibited 8-fold enhancement with PLQY up to 21.9% in aqueous solutions, as shown in Fig. 5c and Table 2. In contrast, BSA modified DMF-CDs also exhibited enhanced emission, while their highest PLQY is just 7.3% in aqueous solutions (Table 2).

It is interesting to find that the multiphoton induced red FL from FA-CDs can be obviously enhanced after combing with BAS in aqueous solutions. As shown in Fig. 5d, e, g, h, under 1150 nm and 1550 nm fs pulse laser excitation, the red emission intensities of FA-CDs and FA-CDs@BSA aqueous solutions were found to increase linearly with the square and cube of laser power, respectively (Fig. S1), indicating the two- and three-photon FL behaviors^6^. The two- and three-photon FL of FA-CDs@BSA aqueous solutions was 15-fold and 12-fold enhancement than that of pure FA-CDs aqueous solutions under 1150 nm fs pulse laser excitation at 3.5 mW and 1550 nm fs pulse laser excitation at 5 mW, respectively. These results indicated that combination with BSA played an important role in protecting FL quenching by water molecules to realize both enhanced single and multi-photon FL from FA-CDs in aqueous solutions.

The combination structure of FA-CDs@BSA were further analyzed. The zeta potential measurements showed that both FA-CDs and BSA were negatively charged with zeta potentials of −31.12 mV and −17 mV, respectively, while the zeta potential value became −21.55 mV of the combination of FA-CDs and BSA (FA-CDs:0.01 mg mL^−1^, BSA:1 mg mL^−1^), indicating that FA-CDs and BSA formed complexes (FA-CDs@BSA) through electrostatic interactions^29^.

The conformational changes of BSA proteins before and after combining with FA-CDs were investigated by circular dichroism spectroscopy. As shown in Fig. S2, the circular dichroism signals of BSA were changed slightly after composing with FA-CDs, indicating no significant conformational changes of BAS in FA-CDs@BSA. Considering that wrapping on FA-CDs could cause significant conformational changes of BAS, it can be inferred that BSA molecules were absorbed on the surface of FA-CDs through electrostatic interactions. The morphology of FA-CDs@BSA was investigated by TEM, as shown in Fig. S3. BSA is a globulin with a Stoke radius of approximately 3.5 nm^30^. The high contrast dots with sizes of about 4.2 nm correspond to FA-CDs, while the low contrast particles with sizes from 20 to 100 nm can be assigned to BAS clusters. It can be seen that all BAS clusters contain several FA-CDs, which are the FA-CDs@BSA. It can be inferred that FA-CDs@BSA are the combination of multiple FA-CDs and BAS molecules with much larger particle sizes, in which BSA molecules are adsorbed on the surface of FA-CDs by electrostatic interactions.

**Fig. 6:**
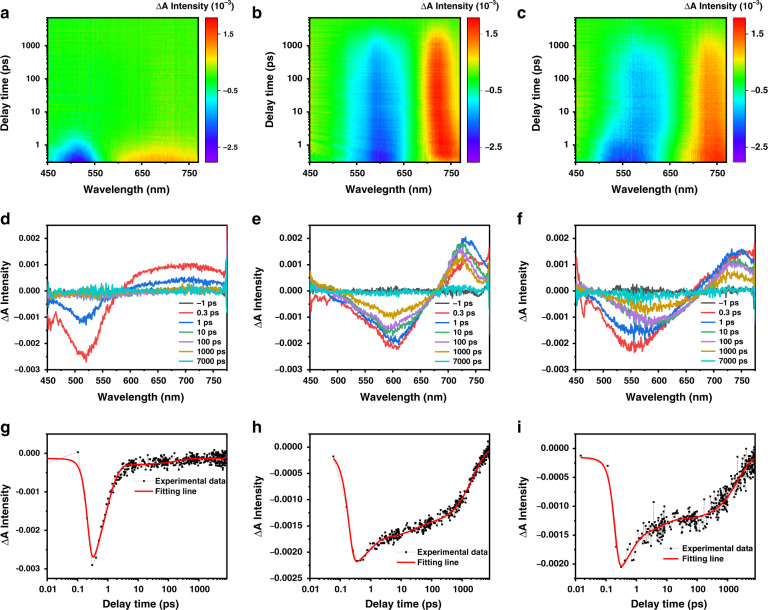
Femtosecond transient absorption (TA) spectroscopy of FA-CDs in water and DMSO, and FA-CDs@BSA in water Two-dimensional pseudo-color map of TA spectra of **a** FA-CDs (0.5 mg mL^−1^) in water, **b** FA-CDs (0.5 mg mL^−1^) in DMSO and **c** FA-CDs@BSA (FA-CDs: 0.5 mg mL^−1^, BSA: 50 mg mL^−1^) in water with a pump wavelength of 400 nm (1 kHz, 100 fs). TA spectra of **d** FA-CDs in water, **e** FA-CDs in DMSO and **f** FA-CDs@BSA in water at indicated delay times. Bleach signal kinetic traces of **g** FA-CDs in water, **h** FA-CDs in DMSO and **i** FA-CDs@BSA in water at pump wavelength of 400 nm and probe wavelengths of 520 nm, 600 nm and 580 nm, respectively. The black squares represent experimental points, while solid lines represent fitting lines

Femtosecond transient absorption (TA) spectroscopy were carried out to explore the excited state dynamics of FA-CDs in water and DMSO, and FA-CDs@BSA in water (Fig. 6). The ground state bleaching (GSB) signals of FA-CDs in water, FA-CDs in DMSO and FA-CDs@BSA in water were observed at 520 nm, 600 nm and 580 nm, respectively, which agree with their steady-state absorption spectra in general. Their positive excited state absorption (ESA) signals were observed at 700 nm, 730 nm and 750 nm, respectively, which exhibited the similar decay lifetimes with their corresponding GSB signals. Comparing the TA kinetic traces of their GSB signals, it is clear that FA-CDs in water show an extremely fast decay within 4.21 ps, while FA-CDs in DMSO show a much a longer lifetime to thousands of ps. The ultra-fast decay of FA-CDs in water can be attributed to the rapid electrons transfer quenching of the excited state by water molecules. After combining with BSA, FA-CDs@BSA in water exhibited a much longer lifetime than that of FA-CDs in water, further demonstrating the combination of BAS can effectively avoid the energy losses in aqueous solutions by preventing the interactions between the C = O groups on FA-CDs and water molecules.

**Fig. 7:**
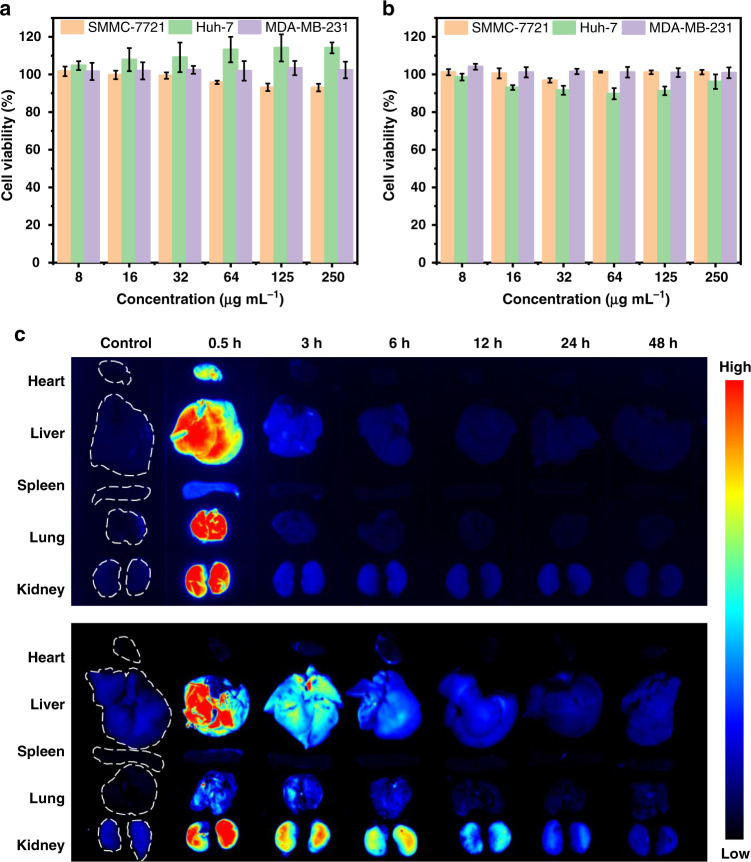
Cell viabilities and biodistribution fluorescence (FL) imaging of FA-CDs and FA-CDs@BSA Cell viabilities of SMMC-7721, Huh-7 and MDA-MB-231 cells after incubation with various containing FA-CDs concentrations in **a** FA-CDs and **b** FA-CDs@BSA (mass ratio of FA-CDs and BSA: 1:100) for 24 h, respectively. The results of three experiments are expressed as means ± standard deviation (SD). **c** FL imaging of the major organs of mice at several time points before and after 0.5, 3, 6, 12, 24 and 48 h intravenous injections of FA-CDs (0.2 mg mL^−1^) (upper) and FA-CDs@BSA (FA-CDs: 0.2 mg mL^−1^, BSA: 20 mg mL^−1^) (bottom) aqueous solutions

The cytotoxicity of FA-CDs and FA-CDs@BSA (mass ratio of FA-CDs and BSA: 1:100) was assessed in SMMC-7721, Huh-7 and MDA-MB-231 cells. As shown in Fig. 7a, b and Fig. S4, FA-CDs and FA-CDs@BSA barely affected cell viability at concentrations containing FA-CDs up to 250 μg mL^−1^; thus, they are of low cytotoxicity.

Due to the red fluorescent properties and low cytotoxicity, we further examined the biocompatibility and biodistributions of FA-CDs and FA-CDs@BSA in mice. Red FL imaging of main organs (heart, liver, spleen, lungs and kidneys) before and after (0.5, 3, 6, 12, 24 and 48 h) intravenous injections of FA-CDs and FA-CDs@BSA aqueous solutions were performed, as shown in Fig. 7c. Based on changes of the red FL signals in the main organs, it can be seen that both FA-CDs and FA-CDs@BSA could be excreted from the mice via liver and kidney in 48 h after intravenous injections. In contrast, FA-CDs@BSA exhibited a longer circulation time than pure FA-CDs, which can be due to their increased particle sizes after combining with BSA.

**Fig. 8:**
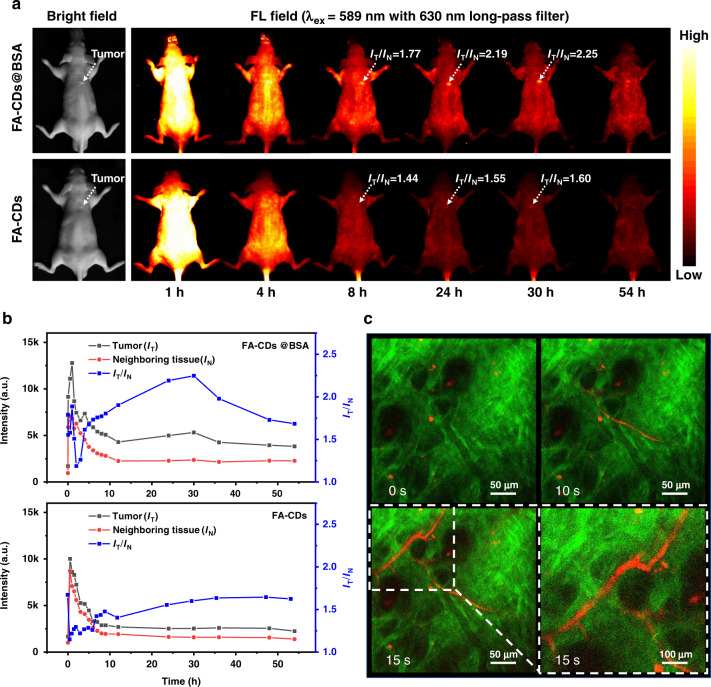
In vivo tumor FL imaging and two-photon FL imaging of FACDs@BSA **a** In vivo tumor imaging pictures of nude mice before and after (1, 4, 8, 24, 30 and 54 h) intravenous injections of FA-CDs@BSA (FA-CDs: 0.2 mg mL^−1^, BSA: 20 mg mL^−1^) and FA-CDs (0.2 mg mL^−1^) aqueous solutions. **b** The FL intensities of tumor and neighboring tissue, and I_T_/I_N_ at several time points before and after intravenous injections of FA-CDs@BSA and FA-CDs aqueous solutions. **c** Two-photon FL images of blood vessels of mouse ear before and after (10 s and 15 s) intravenous injection of FA-CDs@BSA (FA-CDs: 0.2 mg mL^−1^, BSA: 20 mg mL^−1^) aqueous solutions. Dashed lines indicate partial enlargement

The feasibility of in vivo FL tumor imaging of mice using FA-CDs and FA-CDs@BSA were also tested. After intravenous injection of 0.2 mL FA-CDs@BSA aqueous solutions (FA-CDs: 0.2 mg mL^−1^, BSA: 20 mg mL^−1^) or FA-CDs aqueous solutions (0.2 mg mL^−1^) into nude mice with 4T1 tumor, each mouse’s entire body developed a bright red FL over time, as shown in Fig. 8a. At 4 h post injection, red FL intensity was significantly reduced throughout the body, whereas the red FL signal in the tumor region of the FA-CDs@BSA treated mouse could last in much higher intensity for much longer time than that of the FA-CDs treated mouse. As seen in Fig. 8b, after 12 h of injection, FL signal contrast (I_T_/I_N_) of the red FL intensities at the tumor site (I_T_) and the near tissue (I_N_) in the FA-CDs@BSA treated mouse can reached a value higher than 2, which can last for a long observation time window in more than 6 h. In comparison, the FL signal ratio in tumor site of the FA-CDs treated mouse is much smaller in the whole observation period. It can be inferred that combination with BSA can increase the particle sizes of FA-CDs@BSA, which promote longer blood circulation and higher accumulation of FA-CDs@BSA in the tumor site through enhanced permeability and retention (EPR) effect, leading clearly red FL tumor imaging performance.

Considering the significant two-photon red FL of FA-CDs@BSA in aqueous solutions, in vivo two-photon red FL imaging of mouse ear vessels was carried out based on FA-CDs@BSA. Mouse was given intravenous injection of 0.2 mL FA-CDs@BSA aqueous solutions (FA-CDs: 0.2 mg mL^−1^, BSA: 20 mg mL^−1^). Green (506–593 nm) and red (604–678 nm) channel emissions from the mouse ear were collected under 1150 nm fs laser excitation at 4.5 mW. The green signal was the second harmonic signal from 1150 nm fs laser of collagen fibers in the mouse ear. A one-minute video of two-photon red FL imaging for the mouse ear under 1150 nm fs laser excitation was recorded before and after intravenous injection of FA-CDs@BSA aqueous solutions (Video S1). As shown in Fig. 8c, significant two-photon red FL signal from FA-CDs@BSA can be clearly detected in the mouse ear vessels at 15 s post injection. Moreover, after 40 mins of injection, bright two-photon red FL signals can be still clearly observed in the vasculature of the mouse ear (Fig. S5). The above findings show that FA-CDs@BSA can be employed as low cytotoxic and effective red FL probe for in vivo tumor FL imaging and two-photon FL imaging with a long observation time window.

## Discussion

**Fig. 9:**
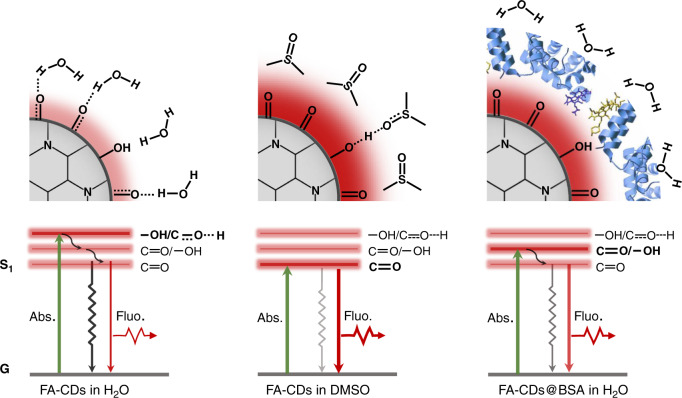
Schematic diagram of possible mechanism Possible surface environments and energy level alignments of FA-CDs in water, DMSO and after combining with BSA in water

Based on the results above, a possible mechanism for the enhanced red emission of FA-CDs in DMSO and their BSA composites in water is proposed in Fig. 9. The surface of FA-CDs is abundant in electron withdrawing groups such as C = O and rare in proton-donating groups such as –OH and –NH, leading to uniform strong electron-withdrawing structure with a main red emission center. In water, C = O groups on FA-CDs directly contact with water molecules through inter molecular hydrogen bonds, which weaken the surface electron-withdrawing ability and cause rapid electrons transfer quenching of the excited state by water molecules, leading to weak red emission. In DMSO, –NH and –OH groups on FA-CDs form strong hydrogen bonds with DMSO molecules, which cause surface deprotonation of FA-CDs and prevent energy dissipation by the vibration of the surface hydroxyl groups, leading to enhanced surface electron-withdrawing environment with enhanced red emission. For FA-CDs@BSA, the surface combined BSA molecules can effectively prevent water molecules contacting with the surface C = O groups, which sustain the surface electron-withdrawing environment with much smaller energy dissipation by water molecules, leading to efficient red emission in aqueous solutions.

To summarize, we developed a facile one-step solvothermal synthesis of red emissive FA-CDs from citric acid and urea in FA without complicated purification procedures. By comparing the optical properties and chemical structures of the solvothermal synthesized samples in different solvents, the high content of electron-withdrawing C = O groups and low content of proton-donating −NH and −OH groups on the surface of FA-CDs account for their pure red emission. After composing with BSA, high PLQY up to 21.9% and significantly enhanced multi-photon red FL were realized in FA-CDs@BSA aqueous solutions. Both FA-CDs and FA-CDs@BSA exhibited very low cytotoxicity and were renally excreted in vivo. More important, clearly in vivo red FL tumor imaging and two-photon red FL imaging of mouse ear vessels were realized via intravenous injection of FA-CDs@BSA aqueous solutions. To our best knowledge, this is the first time of realizing two-photon FL imaging in mammals in vivo based on CDs. We prospect this study can promote the rational design of low-cost strong red/NIR emissive CDs in aqueous system for their high performance biological applications.

## Materials and methods

### Materials

Sigma–Aldrich provided the citric acid, urea, DMF, and DMSO. Acros Organics provided the FA. Beijing Chemical Works supplied the acetone and ethanol. Shanghai Macklin Biochemical Co., Ltd. supplied the acetic acid. A Labconco WaterPros purification system was used to deionize and purify the water. All reagents were of at least analytical grade purity and were used without further purification.

### Synthesis of CDs

CDs were prepared from citric acid (2 g) and urea (4 g) by solvothermal treatment in four different solvents (20 mL) at 160 °C 4 h in a Wattcas autoclave (WP-MSAR-250A). The CDs prepared in four solvents, AcOH, DMK, DMF and FA, are named AcOH-CDs, DMK-CDs, DMF-CDs and FA-CDs, respectively. In detail, the obtained dark brown solutions after solvothermal treatment in AcOH and DMK were packed into dialysis bags (cut-off molecular weight 2000) and dialyzed with ultra-pure water for two days to obtain AcOH-CDs and DMK-CDs, respectively. The obtained dark red-brown solutions after solvothermal treatment in DMF and FA were mixed with 40 mL ethanol, shaken well, and then centrifuged at 10,000 r min^−1^ for 5 min and repeated 2–3 times. The precipitates were freeze-dried to obtain the black product of DMF-CDs and FA-CDs, respectively.

### Synthesis of FA-CDs@BSA

3 mL aqueous suspension of FA-CDs (0.02 mg mL^−1^) was added to 3 mL BSA aqueous solutions of BSA with concentrations of 0.02–6 mg mL^−1^. The mixed solutions were heated at 50 °C for 10 min to give rise FA-CDs and their BSA composites (FA-CDs@BSA) with different composing ratios. For biodistribution analysis, tumor and mouse ear vessels imaging, the FA-CDs@BSA were prepared at a mass ratio of FA-CDs and BSA with 1:100 at FA-CDs concentration of 0.2 mg L^−1^.

### Characterization

The samples’ morphologies were examined using a transmission electron microscope (FEI TecnaiG2F30; 200 kV). Thermo Fisher Scientific’s ESCALAB 250Xi photoelectron spectrometer was used to perform X-ray photoelectron spectroscopy with Mo as the excitation source. A JASCO V-770 spectrophotometer was used to acquire UV-vis absorption spectra. Ocean Optics QE Pro spectrofluorometer was used to measure the PL spectra. An Edinburgh FS5 spectrophotometer was used to measure the PL lifetime and PLQY. The fundamental-frequency 800 nm femtosecond laser pulse was generated by the Coherent Legend regenerative amplifier (100 fs, 1 kHz) which is seeded by a Coherent Vitesse oscillator (100 fs, 80 MHz). An automated optical parametric amplifier (Light Conversion, TOPAS Prime) pumped by the fundamental-frequency 800 nm femtosecond laser pulse was used to obtain the 1150 and 1500 nm femtosecond lasers employed in multiphoton FL. The two-photon and three-photon FL signals were collected by a spectrometer (ANDOR, Shamrock 303i) coupled with CCD (ANDOR, Newton DU920P). An Ultrafast System HELIOS spectrometer in a nondegenerate pump-probe configuration was used to measure TA. The 400 nm pump laser was obtained by propagating the 800 nm fundamental-frequency femtosecond laser pulse through a 0.5 mm thick BBO single crystal.

### In vitro cytotoxicity study

The Alamar Blue assay was performed to assess the cytotoxicity of FA-CDs and FA-CDs@BSA using SMMC-7721, Huh-7, and MDA-MB-231 cells. FA-CDs@BSA are composed at a mass ratio of FA-CDs and BSA with 1:100. Cells were seeded at a density of 5000 cells per well in 96-well plates and incubated overnight. Then the medium was changed with 100 μL fresh medium containing FA-CDs and FA-CDs@BSA with various concentrations. After 24 and 48 h of incubation, the medium was changed with 100 μL fresh medium containing 10% Alamar Blue, and the cells were incubated for a further 3–4 h. The cell viability was determined by measuring the absorbance of the correlated cells at Ex 560 nm/Em 590 nm using a microplate spectrophotometer with the cells only cultured with medium as a control. Three wells were tested in parallel in each experiment.

### In vivo biodistribution imaging

In vivo biodistribution imaging of FA-CDs and FA-CDs@BSA were performed using 6- to 8-week-old Fvb mice. FA-CDs and FA-CDs@BSA aqueous solutions (200 μL) were injected intravenously (tail vein) in mice at various time points (0.5, 3, 6, 12, 24 and 48 h) before mice were sacrificed to obtain organ imaging simultaneously. ChemDoc^TM^ MP Imaging System (Bio-Rad Laboratories, Inc.) was used to acquire the images under 546 nm excitation with a 602/50 nm emission filter. During the experiments, no animal displayed any signs of acute toxicological effects.

### In vivo fluorescent tumor imaging

4T1 cells (5*10^5^ per mouse) were injected subcutaneously into the upper dorsal region of nude mouse to generate the 4T1 tumor mouse model. As the tumor size reached 1–9 mm^3^, these tumor-bearing nude mice were preforming in vivo one-photon FL imaging. The FA-CDs and FA-CDs@BSA (200 μL) were injected intravenously into nude mice with 4T1 xenograft tumors. ANDOR iXon Life 888 Electron Multiplying CCD Cameras (Oxford Instruments) was used to acquire the images with 589 nm excitation laser and 630 nm long-pass filter.

### Two-photon FL blood vessels imaging

Two-photon in vivo angiography based on FA-CDs@BSA was performed on B6-albino female mice. A mouse aged 6 to 8 weeks was anesthetized with avertin (0.25 mg g^−1^) intraperitoneally, and then 200 μL of FA-CDs@BSA aqueous solutions were delivered intravenously. The mouse was put on the microscope stage with one ear attached to the coverslip and the ear was applied with glycerol to minimize optical interference. A one-minute video of two-photon FL imaging for the mouse ear under 1150 nm laser excitation was recorded and FA-CDs@BSA aqueous solutions was injected at 15 s after the start of the recorded video, which is available in the supporting information (Video S1). The femtosecond laser beam was focus onto the ear using a 40X NA = 1.15 water-immersion objective. The laser power at the sample was ≈4.5 mW. Nikon Eclipse Inverted Multiphoton Microscope (A1MP + Eclipse Ti-2E, Nikon instrument Inc., Japan) was used to acquire the images.

## Supplementary information

Video S1-Supporting Movie

Supplementary video description

Supporting Information
