# Supplementary material for: One step synthesis of efficient red emissive carbon dots and their bovine serum albumin composites with enhanced multi-photon fluorescence for in vivo bioimaging
Source: Light Sci Appl. 2022 Apr 27;11:113. doi: 10.1038/s41377-022-00798-5 (PMC9046223; doi:10.1038/s41377-022-00798-5)
Supplement: Supplementary file 3 — Supporting Information [file 41377_2022_798_MOESM3_ESM.docx]

**Supporting Information for**

**One Step Synthesis of Efficient Red Emissive Carbon Dots and Their Bovine Serum Albumin Composites with Enhanced Multi-Photon Fluorescence for *in vivo* Bioimaging**

*Huiqi Zhang^1^, Gang Wang^1^, Zhiming Zhang^2^, Josh Haipeng Lei^3^,* *Tzu-Ming Liu^2^, Guichuan Xing^1^, Chu-Xia Deng**^3^, Zikang Tang^1^, and Songnan Qu ^1, *^*

*^1^ Joint Key Laboratory of the Ministry of Education, Institute of Applied Physics and Materials Engineering, University of Macau, Taipa, Macau SAR, 999078, China.*

*^2^ Institute of Translational Medicine, Faculty of Health Sciences, University of Macau, Taipa, Macau SAR, 999078, China; MoE Frontiers Science Center for Precision Oncology, University of Macau, Taipa, Macau SAR, 999078, China.*

*^3^ Cancer Center, Faculty of Health Sciences, University of Macau, Taipa, Macau SAR, 999078, China; MoE Frontier Science Centre for Precision Oncology, University of Macau, Taipa, Macau SAR, 999078, China.*

*Email:* [*songnanqu@um.edu.mo*](mailto:songnanqu@um.edu.mo)

**Table of Content**

1. **Figure S1.** Plot of logarithm two-photon/three-photon emission intensities versus logarithm the laser power of FA-CDs and FA-CDs@BSA.
2. **Figure S2.** The circular dichroism spectra of FA-CDs and FA-CDs@BSA.
3. **Figure S3.** TEM image of [FA-CDs@BSA.](mailto:FA-CDs@BSA(FA-CDs:0.01mg)
4. **Figure S4.** Cell viabilities for 48 h.
5. **Figure S5.** Two-photon fluorescence image of blood vessels of mouse ear after 40 min intravenous injection of FA-CDs@BSA aqueous solutions.


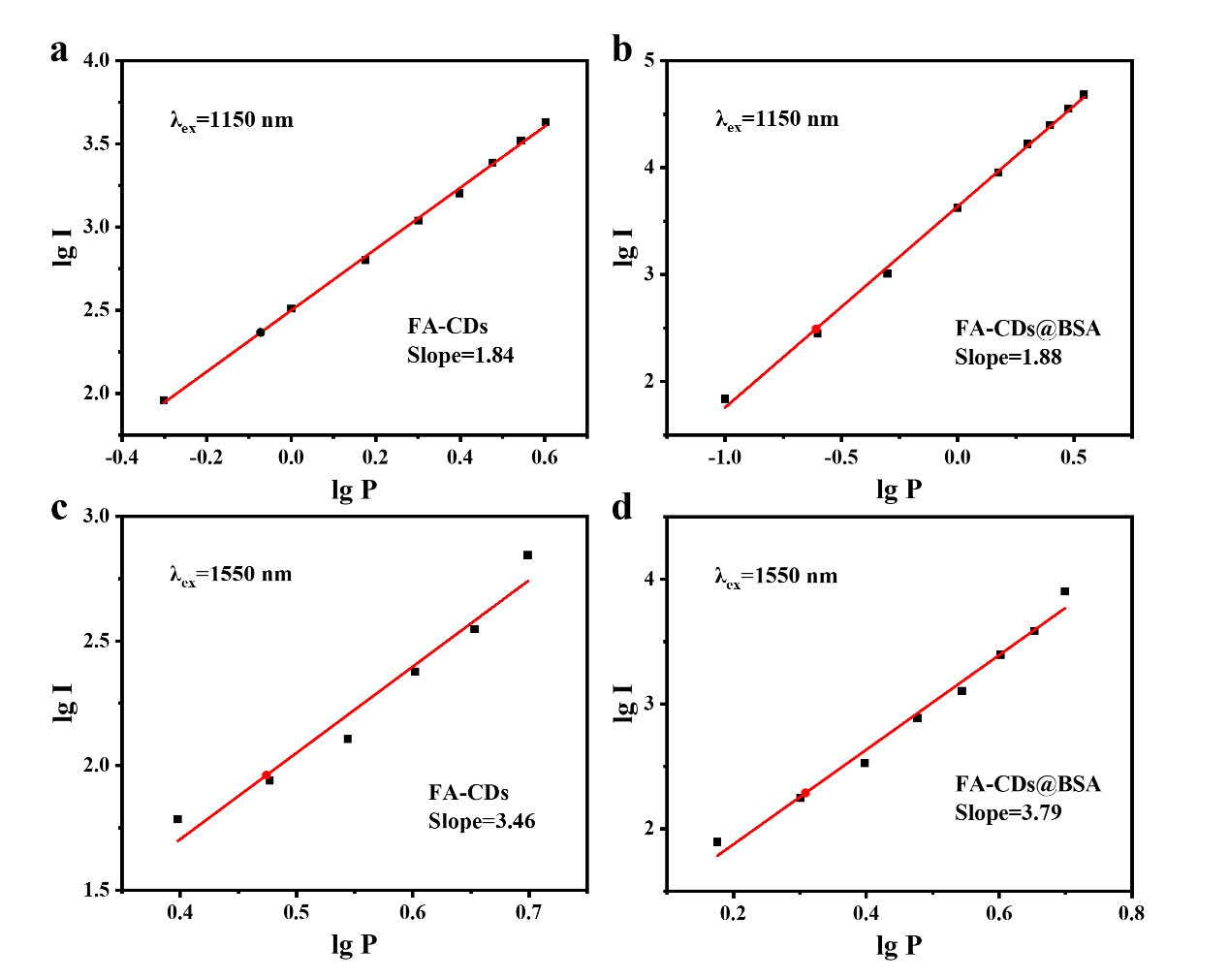


**Figure S1.** Plot of logarithm (**a**)(**b**) two-photon/(**c**)(**d**) three-photon emission intensities versus logarithm the laser power of FA-CDs (0.5 mg mL^−1^) and FA-CDs@BSA (FA-CDs: 0.5 mg mL^−1^, BSA: 50 mg mL^−1^).


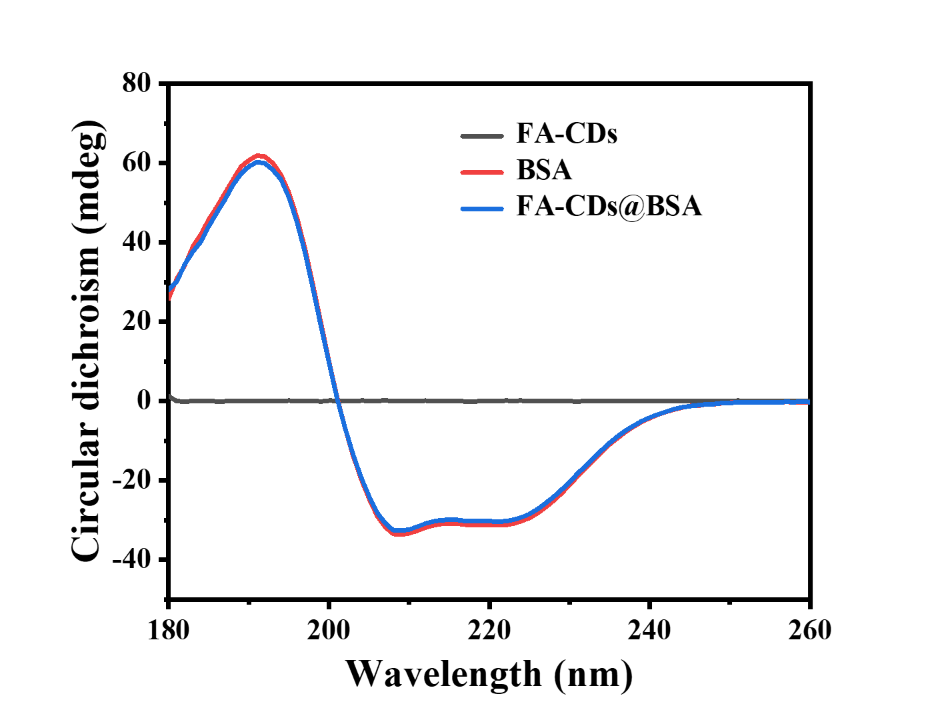


**Figure S2.** The circular dichroism spectra of FA-CDs ([0.002 mg](mailto:FA-CDs@BSA(FA-CDs:0.01mg) mL^-1^), BSA (0.2 mg mL^-1^) and [FA-CDs@BSA (FA-CDs: 0.002 mg](mailto:FA-CDs@BSA(FA-CDs:0.01mg) mL^-1^, BSA:0.2 mg mL^-1^) in aqueous solutions.


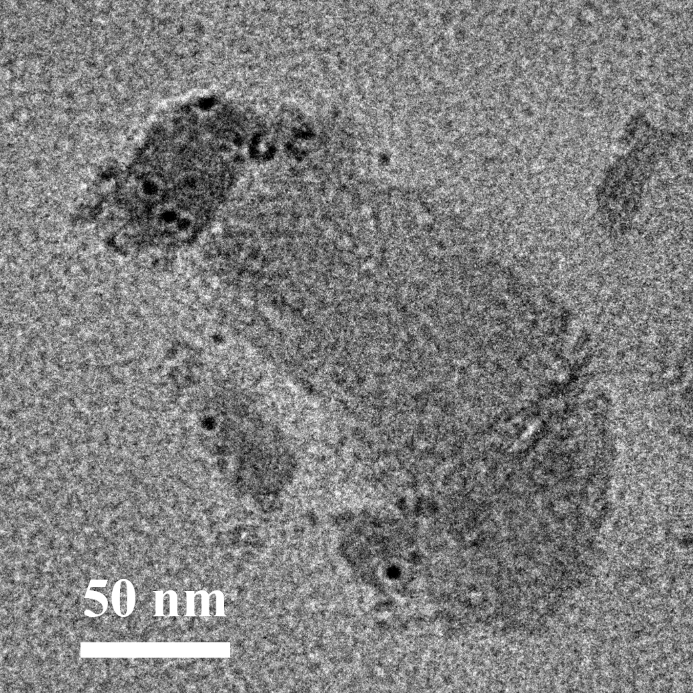


**Figure S3.** TEM image of [FA-CDs@BSA (mass ratio of FA-CDs and BSA: 1:100](mailto:FA-CDs@BSA(FA-CDs:0.01mg)).


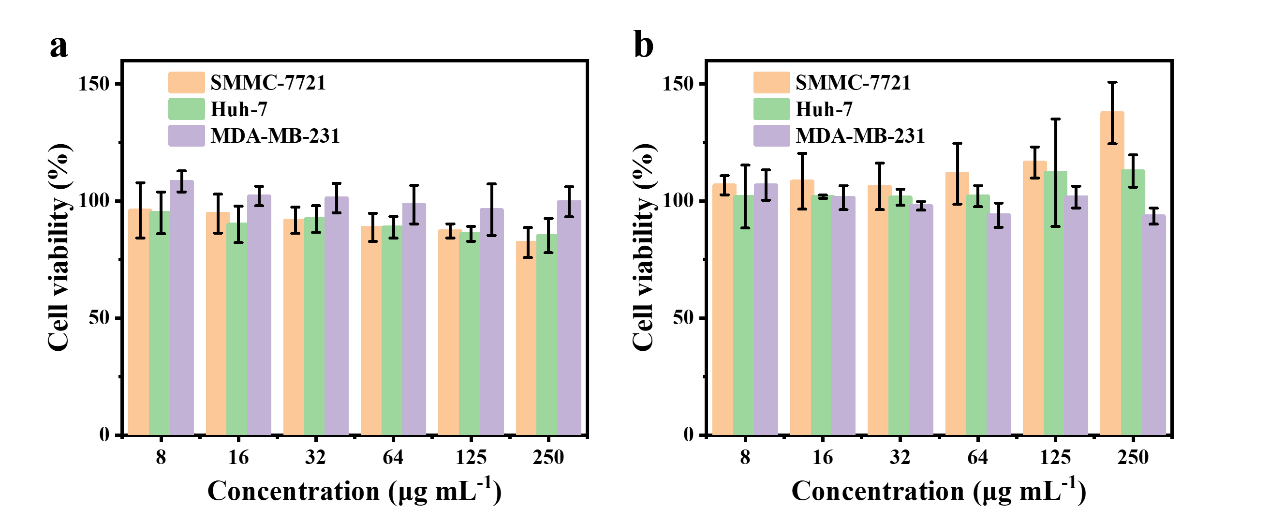


**Figure S4.** Cell viabilities of SMMC-7721, Huh-7 and MDA-MB-231 cells after incubation with various containing FA-CDs concentrations in (**a**) FA-CDs and (**b**) FA-CDs@BSA (mass ratio of FA-CDs and BSA: 1:100) for 48 h, respectively. Data are represented as means ± standard deviation (SD) from three experiments.


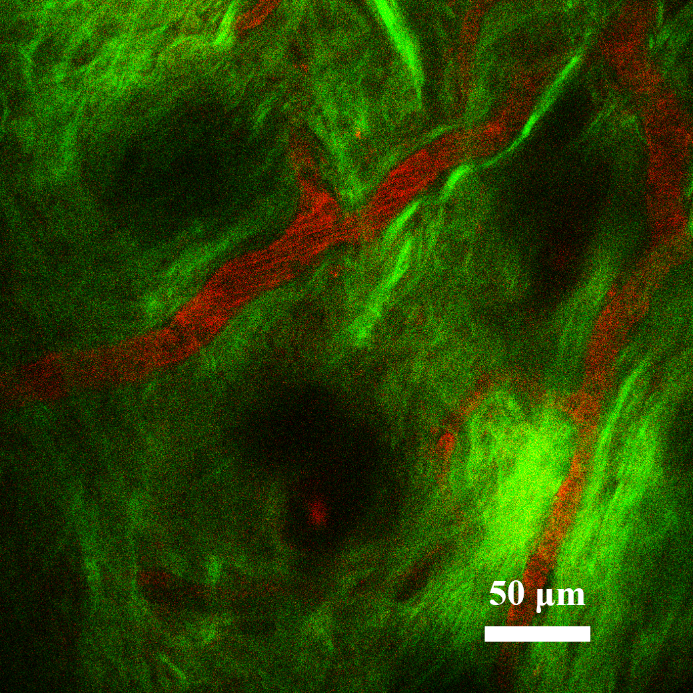


**Figure S5.** Two-photon fluorescence image of blood vessels of mouse ear after 40 min intravenous injection of FA-CDs@BSA aqueous solutions.
